# Data-Driven Clustering Analysis for Representative Electric Vehicle Charging Profile in South Korea

**DOI:** 10.3390/s24216800

**Published:** 2024-10-23

**Authors:** Kangsan Kim, Geumbee Kim, Jiwon Yoo, Jungeun Heo, Jaeyoung Cho, Seunghyoung Ryu, Jangkyum Kim

**Affiliations:** 1 Department of Data Algorithm, LG Energy Solution, Gwacheon 13818, Republic of Korea; kim.ks12000@lgensol.com (K.K.); gbeekim@lgensol.com (G.K.); jiwon.yoo@lgensol.com (J.Y.); jungeund1@lgensol.com (J.H.); jyoungcho@lgensol.com (J.C.); 2 Department of Artificial Intelligence and Robotics, Sejong University, Seoul 05006, Republic of Korea; 3 Department of Artificial Intelligence and Data Science, Sejong University, Seoul 05006, Republic of Korea

**Keywords:** electric vehicle, clustering, data analysis, machine learning, battery

## Abstract

As the penetration of electric vehicles (EVs) increases, an understanding of EV operation characteristics becomes crucial in various aspects, e.g., grid stability and battery degradation. This can be achieved through analyzing large amounts of EV operation data; however, the variability in EV data according to the user complicates unified data analysis and identification of representative patterns. In this research, a framework that captures EV charging characteristics in terms of charge–discharge area is proposed using actual field data. In order to illustrate EV operation characteristics in a unified format, an individual EV operation profile is modeled by the probability distribution of the charging start and end states of charge (SoCs).Then, hierarchical clustering analysis is employed to derive representative charging profiles. Using large amounts of real-world, vehicle-specific EV data in South Korea, the analysis results reveal that EV charging characteristics in terms of the battery charge–discharge area can be summarized into seven representative profiles.

## 1. Introduction

With the rapid growth of the electric vehicle (EV) industry, various studies are being conducted on introducing driver-centered services [1,2,3]. For example, a recommendation service about charging infrastructure could improve efficiency by analyzing driving habits and the physical status of the battery [4,5]. Driving distance prediction based on the user’s driving habits could mitigate energy depletion issues [6] or be utilized to develop insurance products [7]. Basically, these applications are based on the analysis of user patterns from EV operation data. However, obtaining and analyzing large amounts of vehicle-specific, long-term EV operation data is challenging due to various factors, e.g., privacy concerns, limited operation history, and variability in data.

Instead of utilizing direct EV operation data, one branch of research analyzes indirect data related to EV operation. This includes, for example, user surveys [8], interviews [9], GPS data [10], and artificially generated charging behaviors [11]. In terms of EV operation characteristics related to the battery, which is the core resource of EVs, a branch of research utilizes anonymized data from charging facilities. For example, in [12], the representative operating time and charging characteristics were derived based on the factors that affect the user’s charging policies. In [13], EVs were classified into two groups (i.e., personal and commercial use), and group-wise charging/operation patterns were analyzed. A two-stage clustering and entropy-weighted method was proposed in [14]. The authors classified EVs into various groups and analyzed the classification results. Furthermore, the charging behavior of EV users was analyzed from numerous public second-tier charging infrastructure data in [15]. Like the above, many research studies are conducted using data collected at an infrastructure level. In general, these datasets treat one charging session (a single instance of plugging in and charging) as one sample. Furthermore, each session is anonymized; thus, it is difficult to reflect the long-term individual characteristics of the EV. Therefore, analysis of representative patterns is derived from the statistical aspects of the overall EV user pool (e.g., charging frequency and operating tendencies).

Compared to previous research, this study conducts vehicle-specific analyses using long-term operation data from EVs. Specifically, the focus of this paper is on identifying representative patterns in terms of battery charge–discharge areas, which is one of the key factors affecting battery degradation [16].

**Figure 1:**
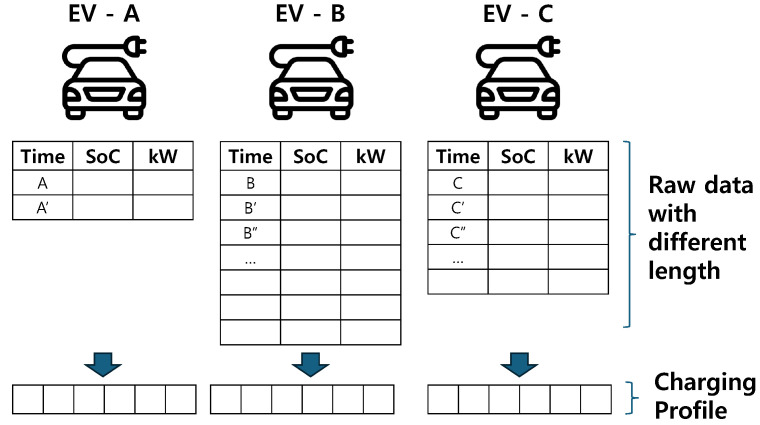
Charging profile representation for unified analysis between EVs.

However, EV operation characteristics vary depending on the owner, and the number of samples collected from each vehicle differs, as illustrated in Figure 1.

This complicates the process of applying a unified analytical framework and makes it difficult to identify consistent patterns across the entire dataset. There are several ways to illustrate EV operation characteristics. A typical approach is utilizing statistical features from EV time-series data, such as average within a day [17,18,19]. By using this approach, it is possible to illustrate what actions are performed at a specific time of day. Based on this, daily charging profiles are adopted to represent changes in the state of battery (e.g., charging load) over a day [20]. In general, daily charging profiles and day-based statistics focus on finding temporal patterns of EV charging load. Therefore, the associated analyses are performed to understand the impact of EV load on the grid based on data collected from charging stations rather than to analyze the impact on the EV itself.

In this research, vehicle-specific data are utilized to characterize battery usage patterns in terms of charge–discharge areas. To this end, the proposed framework models and analyzes the EV charging profile as a joint probability distribution of the start and end states of charge (SoCs). Then, a divergence-based hierarchical clustering algorithm is applied to identify representative profiles of EV groups. The experiments in this paper include an extensive examination of representative patterns across 499 production EVs in South Korea, with data collected through a battery monitoring application (B-Lifecare) developed by LG Energy Solution. The overall contributions of this research can be summarized as follows:

- For a unified analysis of EV battery usage characteristics across varying sample sizes, a novel framework is proposed that models EV charging profiles as a joint probability distribution of the charging start and end SoCs, using data collected from the actual operation of EVs. By utilizing kernel density estimation, the collected samples are transformed within a predetermined time window into a common probability distribution format, enabling consistent data analysis across varying EV operation data.
- To obtain representative profiles from the converted distributions, divergence-based hierarchical clustering is applied. Specifically, the Jensen–Shannon distance is used to measure the similarity between two distributions, aiding in the merging of clusters. The number of clusters is determined using various clustering indices.
- Based on the proposed framework, an extensive analysis is conducted using a real-world EV dataset. By examining data from 499 vehicles collected between 2021 and 2023, with practical interpretations of the clustering results provided by field experts, the findings can be utilized to develop smart services and applications for EVs.

The rest of this paper is organized as follows. First, the overall framework and methodologies are described in Section 2. Section 3 presents experimental results and their analysis. Finally, the discussion and conclusions are given in Section 4.

## 2. Overall Framework

**Figure 2:**
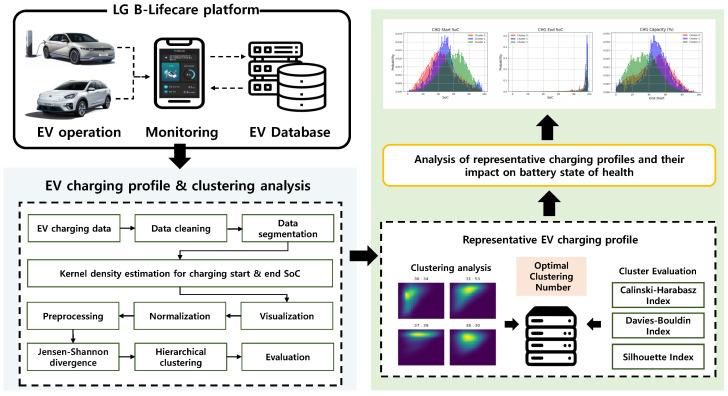
Illustration of representative EV charging pattern analysis framework.

In this research, long-term vehicle-specific EV data are analyzed to derive representative EV charging patterns based on the following analytical framework. As depicted in Figure 2, first, EV driving data (e.g., charging start/end SoC, charging time, amount of energy) are collected from B-lifecare developed by LG Energy Solution.

B-lifecare is an EV battery management solution that provides real-time diagnostics, performance analysis, and predictive maintenance, enabling EV owners to optimize their battery’s lifespan and value. The raw dataset contains time-series EV operation data collected from 499 vehicles during 1 year.

Because the time of vehicle shipment and number of data acquisitions differ for each vehicle and user, the length of data vary according to the EV. Therefore, a common form representing the operation characteristics of EVs is required from varying lengths of time-series data. The status of a battery is often defined in terms of SoC, and the charge–discharge area refers to the general usage range of the battery defined by multiple charging sessions. For a single charging session, a change in SoC can be described as a pair comprising a charging start SoC and end SoC ranging from 0∼1. Then the probability distribution of the pair of start and end SoCs from historical data can be modeled using kernel density estimation. As a result, the area with a high probability characterizes the vehicle-specific charging pattern which is obtained by clustering analysis. For clustering analysis, hierarchical clustering is applied using the Jensen–Shannon (JS) distance as the similarity metric between charging profiles. Hierarchical clustering is one of the fundamental clustering methodologies that allows for intuitive visualization of cluster formation and structure. It begins by exploring the overall structure of the dataset, allowing for the flexible formation of clusters without specifying the number of clusters *k* in advance. In contrast to methods like k-means or Gaussian mixture models, which require *k* to be set beforehand and involve repeatedly recalculating distances for all data points during each update, hierarchical clustering simplifies the comparison of different *k* values without restarting the process for each *k*. This makes it effective for systematically exploring different possible cluster structures.

The similarity of probability distributions is often measured by divergence, so hierarchical clustering is combined with JS distance, which is the square root of JS divergence. After the clustering process, the number of representative profiles is derived based on the comparison of clustering indices, for example, the Silhouette index, Calinski–Harabasz index, and Davies–Bouldin index. Finally, the practical meaning of the representative profiles is illustrated through vehicle operation. The following subsections describe the detailed methodologies employed in the paper.

### 2.1. Data Processing

The raw data consist of vehicle IDs, charging start and end times, and battery charge levels at the start and end of the charging session. There are 499 vehicles in the dataset, and the data are grouped according to the vehicle ID. On average, each EV had 151 charging sessions, with a minimum of 6 sessions and a maximum of 589 sessions. Next, the time-series charging session data are segmented into 3-month intervals, starting from the first month of data collection for each vehicle. This three-month-long data are converted into a charging profile using Gaussian kernel density estimation, described in the next subsection. By using this approach, the data are augmented for clustering analysis while also reflecting seasonal characteristics. After omitting the samples with abnormal values, a total of 1385 profiles are used for clustering analysis.

### 2.2. Kernel Density Estimation for Charging Data

Although each EV has a different number of samples (i.e., charging sessions), its characteristics can be represented by the same shape by modeling the probability distribution. To undertake this, kernel density estimation (KDE) is adopted, which has the advantage of forming a continuous density curve around observed data points [21,22].

For two random variables X,Y and their samples $\left(x_{i},y_{i}\right)$ for i=0,⋯n, two-dimensional KDE $\hat{f}\left(x,y\right)$ is given as

(1) $$\hat{f}\left(x,y\right)=\frac{1}{nh_{x}h_{y}}\sum_{i=1}^{n}K\left(\frac{x-x_{i}}{h_{x}},\frac{y-y_{i}}{h_{y}}\right).$$

Here, *n* is the number of samples (i.e., number of $\left(x_{i},y_{i}\right)$), and $h_{x}$ and $h_{y}$ are the bandwidth which controls the smoothness of kernel for *x* and *y*, respectively. *K* represents the kernel function that utilize a typical Gaussian kernel expressed as follows.

(2) $$K\left(u,v\right)=\frac{1}{2\pi }\exp\left(-\frac{1}{2}\left(u^{2}+v^{2}\right)\right)$$

Since u,v correspond to $\frac{x-x_{i}}{h_{x}}$ and $\frac{y-y_{i}}{h_{y}}$, K(u,v) has a higher value when (x,y) approach $\left(x_{i},y_{i}\right)$. Therefore, observed samples have a larger contribution to the density estimation. Based on the rule of thumb, the selection of bandwidths $h_{x}$ and $h_{y}$ is determined by Scott’s rule. If *n* denotes the number of samples and *d* the dimension of a sample, then Scott’s rule is defined as

(3) $$h=n^{-\frac{1}{d+4}}.$$

In the proposed framework, *X* corresponds to the charging start SoC and *Y* corresponds to the charging end SoC, respectively. By jointly analyzing start and end SoCs and estimating their probability distribution using Gaussian KDE, the characteristics of the battery charge–discharge area can be easily illustrated through a 2D heatmap.

### 2.3. Hierarchical Clustering with Divergence

In order to obtain representative charging profiles from individual EV data, a hierarchical clustering algorithm is applied. Hierarchical clustering organizes tree-structured clusters by iteratively merging two sub-clusters into one. Therefore, starting from *n* clusters of individual samples, the hierarchical clustering algorithm repeatedly combines the closest pairs of clusters until only one cluster remains (i.e., agglomerative clustering), forming a dendrogram that represents the nested grouping relationships among the data points. Furthermore, visualization with the dendrogram helps intuitive interpretation of the clustering results for field experts.

In this regard, the choice of a distance metric is one of the important factors affecting the clustering results. In this research, divergence is utilized instead of Euclidean distance, since the charging profiles are modeled as the probability distribution. Specifically, JS distance, which is the square root of JS divergence, is applied for the distance metric of hierarchical clustering. JS distance calculates the dissimilarity between the two probability distributions in the range 0∼1. For two given probability distributions *P* and *Q*, JS distance is defined by

(4) $$\mathrm{JSDist}\left(P,Q\right)=\sqrt{\frac{1}{2}D\left(P∥M\right)+\frac{1}{2}D\left(Q∥M\right)}.$$

where *D* denotes the Kullback–Leibler (KL) divergence and *M* is the mixture of *P* and *Q*, i.e., $M=\frac{1}{2}\left(P+Q\right)$. If two distributions *P* and *Q* are identical, JS divergence becomes 0, whereas 1 indicates that those two distributions are completely different. KL divergence *D* in Equation 4 also measures the dissimilarity between two probability distributions. However, it does not satisfy symmetry (i.e., D(P∥Q)≠D(Q∥P)), which is essential for the consistency of the clustering algorithm. On the other hand, JS distance satisfies properties of the distance metric including symmetry. By averaging D(P∥M) and D(Q∥M), JS distance gives the same value regardless of the order of *P* and *Q*. Based on the calculation of JS distance, agglomerative clustering is conducted until it forms a single cluster based on Ward’s method. This process forms a dendrogram that represents the agglomerative formulation of clusters. Then, *k* is obtained, which represents clusters by cutting the dendrogram at a height that corresponds to *k* clusters. As a result, the centroid of each cluster (i.e., the average profile) becomes the representative charging profile for EV analysis.

### 2.4. Clustering Index

The fundamental aspect of clustering is organizing several representative groups from many samples. In this regard, the number of clusters affects the analysis of clustering results, and the appropriate number of clusters should be determined by considering both qualitative and quantitative aspects. Qualitatively, it should be easy for human experts to interpret, and quantitatively, clustering indices can be used to assess the goodness of the clustering. The properties of a good cluster can be summarized in two aspects: (a) intra-cluster samples are closely located, and (b) inter-cluster samples are distantly located from each other. There are various metrics that quantify these two characteristics, and three well-known indices are employed to select the number of clusters: the Silhouette index [23], the Davies–Bouldin index [24], and the Calinski–Harabasz index [25].

#### 2.4.1. Silhouette Index (SI)

The Silhouette value of a sample *i* is defined as

$$S\left(i\right)=\frac{b(i)-a(i)}{\max\{a(i),b(i)\}}$$

where a(i) represents the average distance between sample *i* and all other samples in the same cluster. b(i) is the average distance from sample *i* to the nearest neighboring cluster. The Silhouette index (SI) for a dataset is calculated as the mean of the silhouette values of all samples. A smaller a(i) indicates that points within a cluster are close together, while a larger b(i) signifies good separation between clusters. The range of silhouette values is from −1 to 1, where a value close to 1 suggests that the clusters are well defined and appropriate for the data.

#### 2.4.2. Calinski–Harabasz Index (CHI)

The Calinski–Harabasz index is given by

$$CHI=\frac{BCSS}{WCSS}\times \frac{N-k}{k-1}$$

where *N* is the total number of samples and *k* is the number of clusters. The between-cluster sum of squares (BCSS) is the sum of the squared Euclidean distances between each cluster centroid $c_{i}$ and the overall data centroid *c*, each weighted by the number of members in that cluster. The within-cluster sum of squares (WCSS) is the sum of the squared Euclidean distances from each sample to its respective cluster centroid. In this context, a larger BCSS value indicates greater dispersion between clusters, while a smaller WCSS value indicates tighter compactness within clusters. Consequently, a higher CHI value implies that the clustering is well defined and appropriate.

#### 2.4.3. Davies–Bouldin Index

The Davies–Bouldin index is computed as

$$DBI=\frac{1}{k}\sum_{i=1}^{k}\max_{j\neq i}\left(\frac{S_{i}+S_{j}}{d(c_{i},c_{j})}\right)$$

where *k* is the number of clusters and $S_{i}$ is the average distance of samples in cluster *i* to their centroid $c_{i}$. $d(c_{i},c_{j})$ is the distance between centroids of clusters *i* and *j*. Therefore, $S_{i}+S_{j}$ represents dispersion within clusters and $d(c_{i},c_{j})$ represents the separation of different clusters. For a given cluster *i*, the maximum ratio of these two values for i≠j indicates the case of a poorly distinguished cluster. Consequently, a lower average value suggests that the clustering has been performed appropriately.

In the experiment, the range of *k* is set from 2 to 30 to allow for the explainability of the results by human experts. All indices are primarily calculated using Euclidean distance, which is commonly employed in many clustering algorithms. Additionally, the SI result is compared based on the Jensen–Shannon distance to better align with the proposed methodology.

## 3. Experiments and Analysis

In this section, analysis results are given using EV field data from South Korea. Data are composed of the operation data from 499 EVs from September 2021 to October 2023. First, the target number of clusters, *k*, is determined based on clustering indices, and the charging characteristics of *k* representative profiles are analyzed considering the industrial implications.

### 3.1. Determining the Number of Clusters

**Figure 3:**
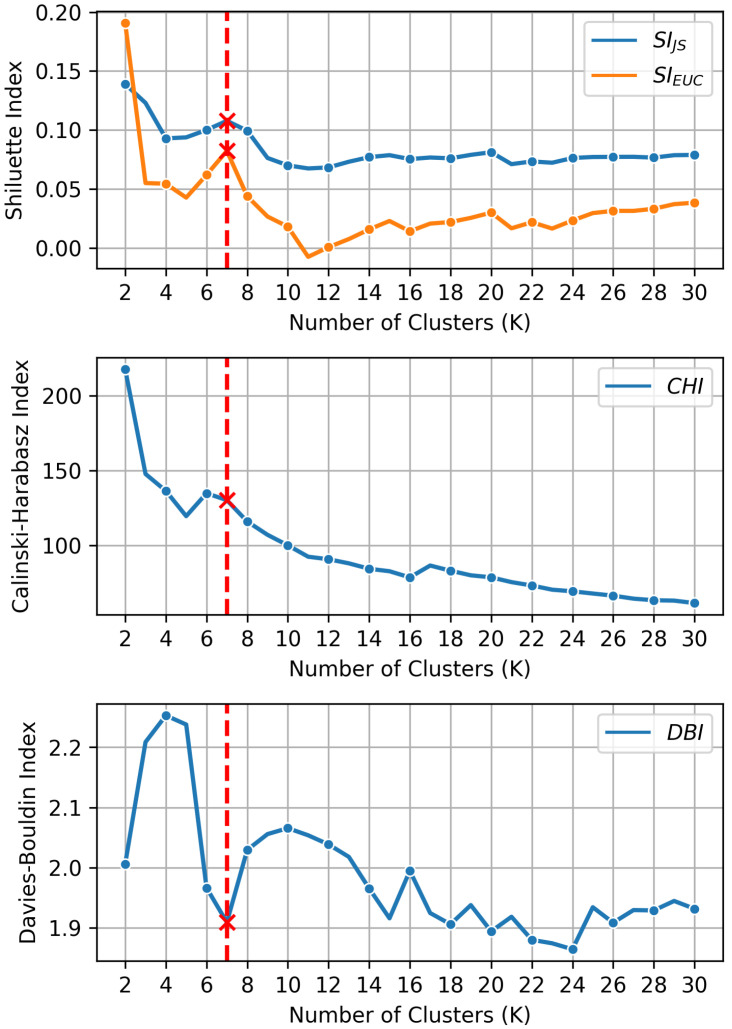
Clustering index (red line in the figure) according to the number of clusters *k*.

Clustering indices on EV field data are evaluated across a range of cluster sizes, *k*, from 2 to 30. Figure 3 illustrates the performance of four different clustering indices for the proposed algorithm, where the *x* axis corresponds to the number of clusters *k* and the *y* axis represents the values corresponding to *k*: SI with JS distance and Euclidean distance, CHI, and DBI. According to the respective formulations of these indices, higher values of SI and CHI and a lower value of DBI indicate more effective clustering (i.e., better separation between clusters and tighter cohesion within clusters). Overall, the graphs show a decreasing trend as the number of clusters *k* increases, leading to conflicting interpretations among the indices. Therefore, the number of clusters, *k*, is set to 7, as it locally satisfies the criteria of a low DBI value, along with high SI and CHI values.

### 3.2. Analysis of Clustering Results

**Figure 4:**
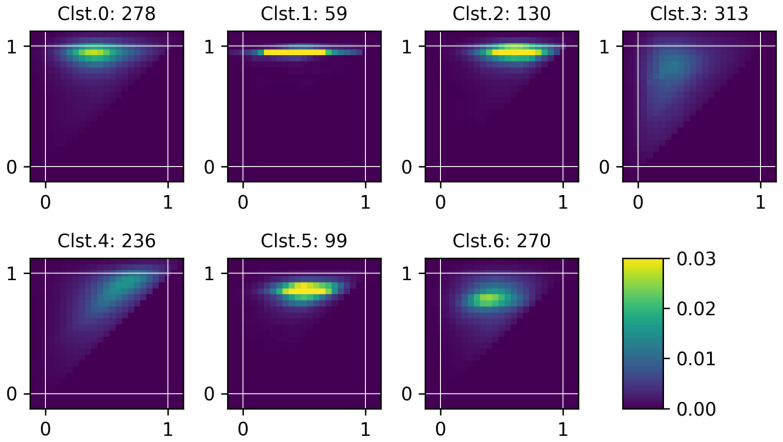
Illustration of representative charging profiles. The label at the top of each image indicates the cluster index and number of members. *x* axis corresponds to start SoC and *y* axis corresponds to end SoC.

After the clustering based on the proposed framework, seven representative charging patterns (i.e., cluster average profiles) are obtained and illustrated in Figure 4. Each representative profile is shown in the form of a 2D heatmap where the average probability is depicted with color. *x* and *y* axes correspond to charging start SoC and end SoC, respectively. Note that the lower-left corner indicates an empty battery state and the upper-right corner indicates a full battery state. The boundary area of the heatmap corresponds to margins that exceed the 0 to 1 SoC range, which is intended for better visualization. First, clusters 0, 1, 2, 5, 6 show a relatively strong tendency in charging patterns compared to clusters 3 and 4. For example, cluster 1 tends to fully charge the battery regardless of charging start SoC. This characteristic appears in the form of distinct horizontal line patterns. Clusters 0 and 2 have relatively dispersed horizontal segments compared to cluster 1, indicating that charging sessions end with varying SoCs. Clusters 3 and 4 exhibit more widely dispersed patterns compared to the others. Therefore, these clusters show no dominant charging behavior, suggesting that the battery is used with a wide range of SoCs. However, differences can be found between clusters 3 and 4 as follows. First, cluster 3 shows a vertically spread pattern on the left side. This can be interpreted as users tending to start charging when the SoC level is below 50% and end charging uniformly. Cluster 4 shows a diagonally dispersed pattern concentrated on the right side, suggesting that the batteries are partially charged. In other words, since the diagonal line across (0,0) and (1,1) coordinates indicates a y=x graph, diagonal segments in the heatmap represent that the difference between end SoC and start SoC is small. Clusters 5 and 6 have similar patterns, having dispersed oval segments around 0.3∼0.7 in the start SoC and 0.8∼0.9 in the end SoC. This type of user prefers starting charges at a moderate SoC and ending at a high but not full SoC, possibly indicating a preference for maintaining battery health and operational readiness. Next, individual charging profiles are compared with the cluster center to assess their similarity to the representative pattern.

**Figure 5:**
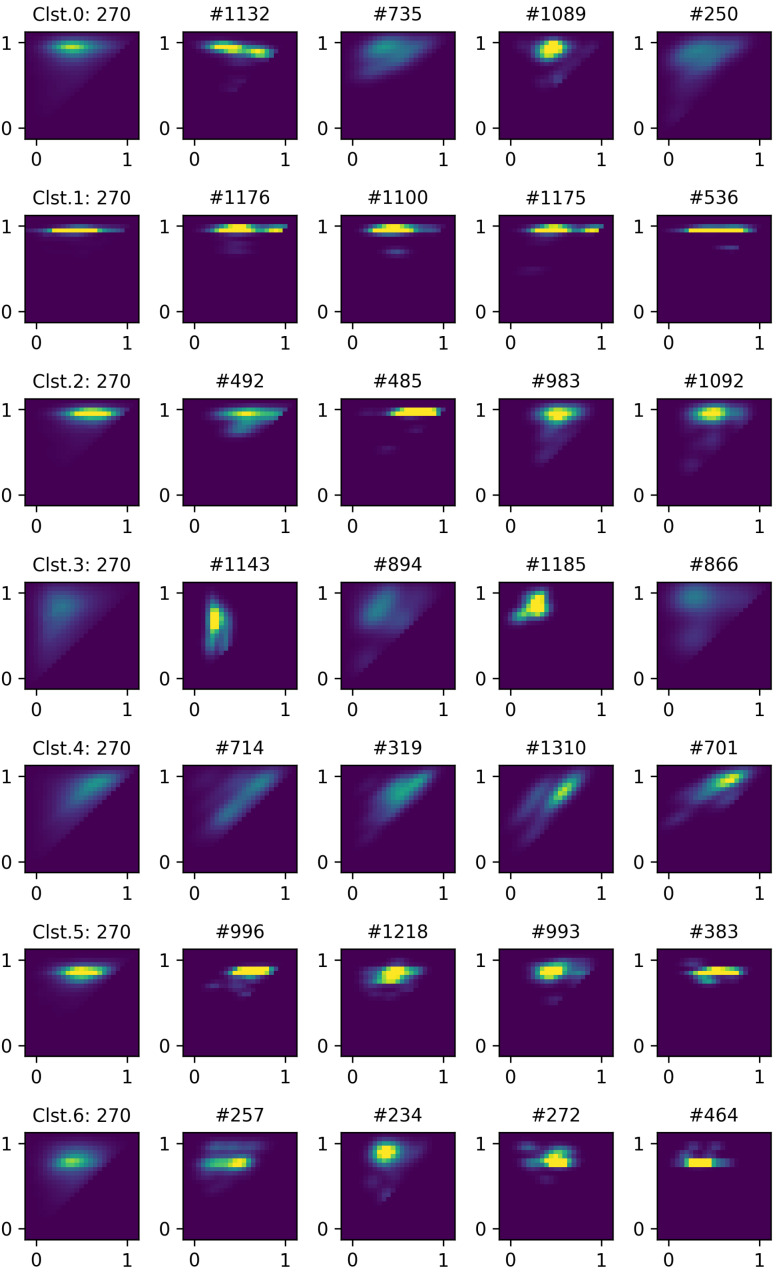
Illustration of cluster centers (first column) and their cluster members. The number with # indicates the profile index.

Figure 5 shows randomly selected members in each cluster. Heatmaps in the first column are representative charging profiles (i.e., cluster center), and the remaining columns are heatmaps of the members in the corresponding cluster. As can be seen, distinctive patterns can be found in a sample in each cluster that matches the above interpretation of representative profiles. For example, members in cluster 1 tend to fully charge the battery regardless of start SoC. Members of cluster 3 (e.g., # 1143, # 1185) show a pattern of starting from a low SoC and charging to a varying SoC level. Additionally, while the representative profiles of clusters 3 and 4 may appear similar in some aspects, the members of these clusters exhibit more distinct pattern differences. Through the visual comparison of representative profiles and members, it can be confirmed that the proposed pipeline works qualitatively well.

The characteristics of each cluster are further examined with statistical features given in Table 1. Table 1 summarizes the statistical characteristics of each cluster including the number of profiles and sessions, the average and the standard deviation of charging start SoC, end SoC, and their difference (i.e., charging amount), respectively. There are 1385 profiles derived from total 66,116 charging sessions. Cluster 3 accounts for the highest proportion at 22.6%, followed by cluster 0 at 20.1%. The top four clusters (0, 3, 4, 6) account for almost 80% of the total profiles. On the other hand, cluster 1 shows the lowest proportion at 4.3%, followed by cluster 5 at 7.1%. This analysis result could be used as a reference indicator for charging infrastructure operators or electric power operators to build and operate the entire system.

Cluster-wise statistics support the quantitative verification of patterns observed in the heatmaps of the representative profiles. Clusters 0, 1, and 2 show horizontal segments in the upper part of the image, indicating that the charging session ends with high SoCs. In line with this, the average end SoC of these clusters exceeds 90% compared to the other clusters. Specifically, Cluster 1 has the highest end SoC with the lowest standard deviation. Cluster 3 has the highest standard deviation in the end SoC, which is illustrated as a vertical segment in the heatmap. A diagonal segment in cluster 4 appears as the lowest average charging amount. Clusters 5 and 6 have oval segments slightly below the horizontal segments of clusters 0, 1, and 2. Therefore the average end SoCs of clusters 5 and 6 are lower than those of clusters 0, 1, 2, respectively.

The distribution of charging amounts and the battery state at the end of each charging session for each cluster were further examined.

**Figure 6:**
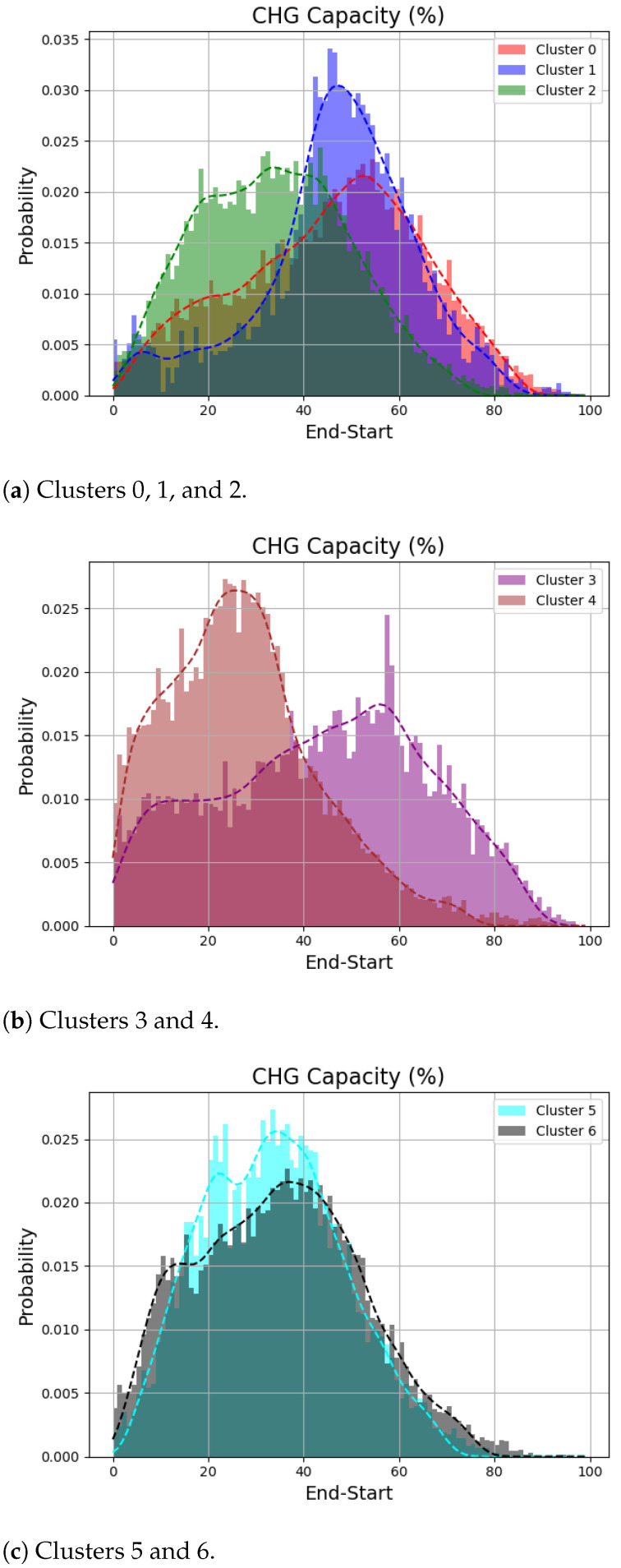
Distributions of charging amount among the clusters.

First, Figure 6 shows empirical probability distributions of the battery charging amounts per charging session according to each cluster. Battery charging amounts are defined as the difference between the charging end SoC and start SoC. As can be seen in the figure, the differences in distribution for clusters 0 to 4 are well illustrated through histograms. Specifically, while the representative patterns of clusters 3 and 4 are less distinct, the actual charging amounts show different distributions. Clusters 5 and 6 show a similar distribution in terms of charging amount. Based on this, regions with a high prevalence of vehicles exhibiting the cluster 4 pattern are anticipated to require extensive charging infrastructure.

**Figure 7:**
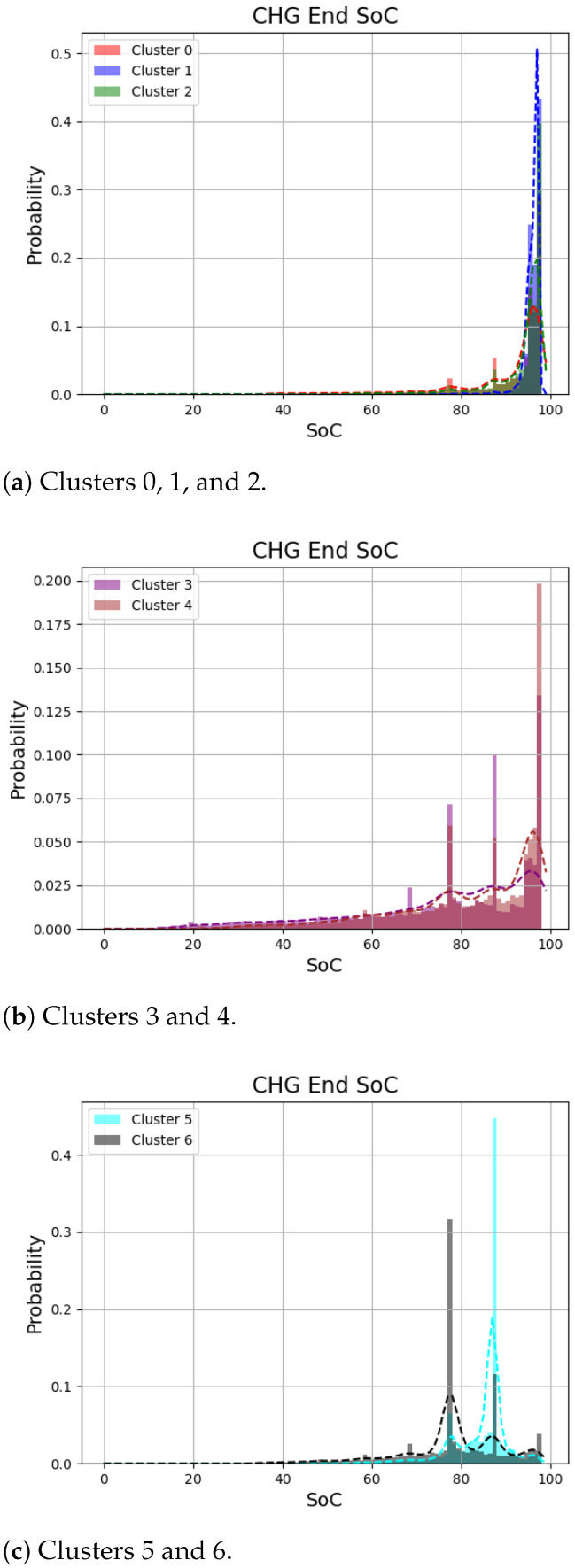
Distributions of charging end SoC according to the cluster.

Next, Figure 7 presents the histogram of charging end SoC for each cluster. For clusters 0∼2, the charging session ends with more than 90% SoC, indicating the EVs in these cluster tend to terminate charging when the battery is fully charged. This is also evident from the high average value of the end SoC shown in Table 1. Similarly, for clusters 3 and 4, while there is a general tendency to terminate charging when fully charged, a significant portion also ends charging at lower levels around 80%. Finally, clusters 5 and 6 tend to terminate charging at certain levels and do not usually fully charge the battery. Given that the average charging amount for clusters 5 and 6 is similar, it can be inferred that the starting SoC value for cluster 5 will be higher compared to cluster 6.

The analysis results can be used to plan EV infrastructure and support battery management. For instance, predicting EV charging power consumption can assist in infrastructure planning, while customized services for battery management can also be offered.

Based on the previous analysis, regions requiring more extensive charging infrastructure can be identified by understanding the charging patterns of different clusters. For instance, regions with a high prevalence of vehicles exhibiting the cluster 4 pattern will need more robust charging facilities due to their frequent charging behavior. Moreover, understanding the tendencies of clusters 5 and 6 to terminate charging at certain levels rather than fully charging can help in optimizing the placement and capacity of charging stations to meet specific needs, thereby enhancing the efficiency of the overall EV network management.

## 4. Conclusions

In this paper, a clustering analysis framework is presented for deriving representative EV charging profiles. In conventional research, various methods have been proposed to achieve representative charging and driving patterns of EVs, but they often have limitations, such as only considering vehicle operating characteristics over short periods or relying on de-identified vehicle data. In contrast, the proposed framework uses vehicle-specific long-term operation data to capture EV charging characteristics and analyze representative patterns in terms of charge–discharge area. To achieve this, Gaussian kernel density estimation and a hierarchical clustering algorithm were utilized. Through this method, seven representative charging profiles were derived, with their distinctive patterns visualized through heatmaps.

Moreover, with vehicle data collected over a longer period of time, the analysis is expected to reflect the seasonality of vehicle operations by extending the data analysis period. Additionally, this method allows for the integration of various multi-modal data, potentially leading to new insights that were not previously explored.

Since the analysis results focus on the variation in SoC in the vehicles, it can be utilized in various ways. For charging infrastructure, the proposed research findings can help specify the appropriate size of charging devices [26]. Additionally, for power system operators, these results can suggest efficient network management methods by predicting regional power demand [27,28].

## Figures and Tables

**Table 1:** Cluster-wise statistics of EV charging data.

Clst.	Profiles		Sessions		Start SoC		End SoC		Chg. Amt.	
					Avg.	Std.	Avg.	Std.	Avg.	Std.
0	278	(20.1%)	14,264	(21.6%)	43.8	(18.2)	89.5	(13.0)	45.6	(19.7)
1	59	(4.3%)	2524	(3.8%)	48.7	(17.1)	95.6	(3.6)	46.9	(17.0)
2	130	(9.4%)	8707	(13.2%)	58.7	(16.2)	92.7	(8.4)	34.0	(16.3)
3	313	(22.6%)	9180	(13.9%)	32.0	(18.3)	76.0	(20.6)	44.0	(22.3)
4	236	(17.0%)	11,398	(17.2%)	54.2	(20.3)	81.4	(16.9)	27.3	(16.5)
5	99	(7.1%)	6333	(9.6%)	50.7	(14.7)	84.2	(7.4)	33.5	(15.0)
6	270	(19.5%)	13,710	(20.7%)	42.9	(15.6)	77.5	(12.4)	34.6	(17.4)

## Data Availability

Data sharing is not applicable to this article.
